# Candidate Strategies for Development of a Rapid-Acting Antidepressant Class That Does Not Result in Neuropsychiatric Adverse Effects: Prevention of Ketamine-Induced Neuropsychiatric Adverse Reactions

**DOI:** 10.3390/ijms21217951

**Published:** 2020-10-26

**Authors:** Motohiro Okada, Yasuhiro Kawano, Kouji Fukuyama, Eishi Motomura, Takashi Shiroyama

**Affiliations:** Department of Neuropsychiatry, Division of Neuroscience, Graduate School of Medicine, Mie University, Tsu 514-8507, Japan; 318d010@m.mie-u.ac.jp (Y.K.); k-fukuyama@clin.medic.mie-u.ac.jp (K.F.); motomura@clin.medic.mie-u.ac.jp (E.M.); takashi@clin.medic.mie-u.ac.jp (T.S.)

**Keywords:** N-methyl-D-aspartate, schizophrenia, mood disorder, L-glutamate, GABA, catecholamine

## Abstract

Non-competitive N-methyl-D-aspartate/glutamate receptor (NMDAR) antagonism has been considered to play important roles in the pathophysiology of schizophrenia. In spite of severe neuropsychiatric adverse effects, esketamine (racemic enantiomer of ketamine) has been approved for the treatment of conventional monoaminergic antidepressant-resistant depression. Furthermore, ketamine improves anhedonia, suicidal ideation and bipolar depression, for which conventional monoaminergic antidepressants are not fully effective. Therefore, ketamine has been accepted, with rigorous restrictions, in psychiatry as a new class of antidepressant. Notably, the dosage of ketamine for antidepressive action is comparable to the dose that can generate schizophrenia-like psychotic symptoms. Furthermore, the psychotropic effects of ketamine precede the antidepressant effects. The maintenance of the antidepressive efficacy of ketamine often requires repeated administration; however, repeated ketamine intake leads to abuse and is consistently associated with long-lasting memory-associated deficits. According to the dissociative anaesthetic feature of ketamine, it exerts broad acute influences on cognition/perception. To evaluate the therapeutic validation of ketamine across clinical contexts, including its advantages and disadvantages, psychiatry should systematically assess the safety and efficacy of either short- and long-term ketamine treatments, in terms of both acute and chronic outcomes. Here, we describe the clinical evidence of NMDAR antagonists, and then the temporal mechanisms of schizophrenia-like and antidepressant-like effects of the NMDAR antagonist, ketamine. The underlying pharmacological rodent studies will also be discussed.

## 1. Introduction

Using N-methyl-D-aspartate/glutamate receptor (NMDAR) inhibiting treatment for mood disorders has been a fundamental discussion in psychiatry and psychopharmacology, since esketamine (a racemic enantiomer of ketamine), a noncompetitive NMDAR antagonist, was approved, although with rigorous restrictions, by the Food and Drug Administration (FDA) and the European Medicines Agency (EMA) in 2019 for the treatment of antidepressant-resistant depressive disorders [1,2]. It is well known that approximately two-thirds of depressed patients fail to achieve an adequate response to first-line pharmacotherapy using conventional monoaminergic antidepressants, such as selective serotonin reuptake inhibitors (SSRI) and serotonin norepinephrine reuptake inhibitors (SNRI), and ultimately as many as one-third of patients remain unwell even after several adequate trials of antidepressants [3]. Furthermore, available medications, such as monoaminergic antidepressants and psycho-behavioural therapies, require more than several weeks for beneficial effects to occur [3]. The delay of conventional monoaminergic antidepressants and psycho-behavioural therapies is one of the major drawbacks to current therapies for major depressive disorder, and faster-acting antidepressants are needed for patients at risk of suicide [4]. Numerous clinical trials have demonstrated that ketamine, a non-competitive NMDAR antagonist, could evoke a rapid onset (within several hours) and shorter sustained (lasting up to 7 days) antidepressive action [5,6,7,8]. Additionally, although monoaminergic antidepressants exhibit limited effectiveness against anhedonia and suicidal ideation of mood disorders, ketamine has also been shown to have distinct and independent anti-suicidal effects in patients with mood disorders [9,10]. Therefore, ketamine holds potential to improve monoaminergic antidepressant-resistant dysfunction of emotional perception/cognition in mood disorders with rapid action. Ketamine comprises two racemic enantiomers, arketamine and esketamine [11,12]. Esketamine (Ki = 0.3~0.7 *μ*M) is a potent antagonist of NMDAR, more so than arketamine (Ki = 1.4~2.6 *μ*M) [11,12]. Similar to the binding affinity, the anaesthetic effect of esketamine is more potent than racemic-ketamine and arketamine [13].

In spite of these clinical advantages of ketamine, both clinical and preclinical studies have established that non-competitive NMDAR antagonists, such as phencyclidine, ketamine, and dizocilpine (MK801), contribute to the pathophysiology of schizophrenia, as they produce schizophrenia-like positive/negative symptoms and cognitive impairments in healthy individuals and experimental animal models, as well as exacerbating the psychotic symptoms of patients with schizophrenia [14,15,16,17,18,19,20,21,22,23,24]. Notably, the ketamine dosage for antidepressant action (0.5 mg/kg) is comparable to that for the generation of psychotic symptoms in healthy volunteers [24]. These findings suggest that ketamine probably both improves emotional/mood states in depression and aggravates perception/cognition in healthy individuals. Therefore, to develop a novel strategy for the treatment of antidepressant-resistant depression without neuropsychiatric adverse effects, the present review is focused on the complicated links between NMDAR and the pathophysiology of schizophrenia and depression.

## 2. Overview of NMDAR

Although NMDAR is characterised as a cation channel-containing receptor, it possesses unique voltage-dependent and substance-sensitive characteristics. Interestingly, activation of NMDAR requires binding of not only glutamate but also D-serine/glycine [25,26,27,28,29]. Furthermore, during the resting stage, glutamate binding to NMDAR cannot induce cation inflow through its cation channel, because the cation channel in NMDAR is blocked by Mg^2+^ and Zn^2+^ caps via specific binding sites [28]. Depolarisation (higher than −20 mV) of the plasma membrane repels Mg^2+^ and Zn^2+^ from the cation channel pore, resulting in the voltage-dependent inflow of Na^+^ and Ca^2+^, and outflow of K^+^, through the cation channel in NMDAR [28]. Notably, the resting membrane potential of GABAergic interneurons is more positive (−50~−60 mV) than those of monoaminergic and glutamatergic neurons (−65~−75 mV) [30,31]. Therefore, at the resting stage, the major targets of NMDAR are GABAergic transmissions rather than monoaminergic or glutamatergic transmissions [29,32,33,34,35,36,37,38,39,40,41,42].

It is well known that NMDAR is a hetero-tetramer receptor comprising three distinct subunits, GluN1, GluN2 and GluN3. The GluN1 subunit exhibits eight splicing variants; GluN1-1a,b, GluN1-2a,b, GluN1-3a,b and GluN1-4a,b (GluN1-1a is the predominant expression in the brain) [43]. The GluN2 subunits subfamily is subdivided into GluN2A, GluN2B, GluN2C and GluN2D subunits, but the GluN3 subunit subfamily is subdivided into GluN3A and GluN3B subunits [25,26,27,28]. The NMDAR cation channel is formed by two GluN1 subunits, and either two GluN2 subunits or a combination of GluN2 and GluN3 subunits [25,26,27,28]. The cation channel pore of predominant functional NMDAR (classical NMDAR) is formed by a combination of the GluN1 dimer and the GluN2 dimer [44]. Mg^2+^, Zn^2+^ and the non-competitive NMDAR antagonists, ketamine, MK801, amantadine and memantine, bind the pore region [27]. Activation of NMDAR requires binding of glutamate to GluN2 together with binding of D-serine/glycine to GluN1. They assemble with GluN1 and GluN2 (A–D) subunits to form tri-heteromeric NMDARs. GluN3 subunits are the newest members of the ionotropic glutamate receptor subunit family, and their detailed functional role remains elusive [45,46]. NMDAR-containing GluN3A seems to counteract some of the well-known functions of classical NMDARs (GluN1–GluN2 di-heteromers) in long-term plasticity and synapse development [45,46].

## 3. Clinical Findings

### 3.1. NMDAR Expression in the Central Nervus System of Patients with Depression and Schizophrenia

It has been established that the expression level of the mRNA of GluN2B is one of the biomarkers of suicide [47]. Additionally, polymorphisms of the GluN2B gene (*GRIN2B*) have been considered to be prediction factors of antidepressant-resistant depression [48,49]. Epigenetic studies found hypermethylation of gene bodies of GluN1 and GluN2A in depression [50,51]. Methylation of the promotor generally downregulates expression of mRNA production, whereas methylation of the gene body increases expression of gene production [52]. Therefore, the hypermethylation of the gene body of GluN1 and GluN2A is possibly due to increased expression of these subunits [53]. Several post-mortem studies of patients with major depression provided fundamental evidence of the pathophysiology of depressive disorders in the cortex and locus coeruleus (LC). Two post-mortem studies reported increased expression of GluN2B and GluN2C in the LC of depressed patients [54,55]. Contrary to the LC, great variability in NMDAR expression in the frontal cortex has been found. In the dorsolateral prefrontal cortex, GluN2B expression was higher in patients with depression who committed suicide when compared to those who did not [47]. GluN1 expression in the prefrontal cortex of patients with depression was almost equal to healthy individuals [56,57], whereas the expression of GluN1 carrying the C1 cytosolic segment was increased [56]. In contrast, reduced expression of GluN2A and GluN2B was seen in the prefrontal and perirhinal cortices [57,58], whereas increased expression of GluN2A was found in the amygdala [59]. Therefore, the results obtained from post-mortem studies suggest consistent increased NMDAR expression in the LC of depressed individuals; however, the expression abnormalities of NMDAR in the frontal cortex are far from consistent, which is possibly due to variations in the target brain regions examined or the methodological procedures.

Reduced GluN2C expression in the cortex of schizophrenics seemed to be consistent evidence; however, meta-analyses could not detect the statistically significant differences in cortical expressions of GluN2A, GluN2B or GluN2D in schizophrenics [60,61,62]. Expression of GluN1 mRNA was also reduced in the prefrontal cortex and hippocampus of schizophrenics [62,63,64,65]. Interestingly, reduction in the density of both postsynaptic protein PSD-95 and downstream signalling associated with NMDAR was displayed in schizophrenia [62,66]. A selective single-photon emission tomography study demonstrated that intravenous administration of ketamine reduced NMDAR in the human brain, including the thalamus and middle inferior frontal cortex of healthy individuals [67]. In particular, reduced NMDAR binding density in the middle inferior frontal cortex significantly correlated with a negative Brief Psychiatric Rating Scale score [67]; however, ketamine-induced reduction in NMDAR expression was not observed in the hippocampus [67].

These findings suggest that enhanced and reduced transmission associated with NMDAR possibly occurs in the brain of individuals with depression and schizophrenia, respectively. However, as aforementioned, care must be taken, since at least some of these changes are observed in different psychiatric conditions and/or possibly induced by medications, and, by no means, are compelling, as long as discrepant results have been observed.

### 3.2. Clinical Pharmacological Findings of NMDAR in Depression and Schizophrenia

Both of the non-competitive NMDAR antagonists, phencyclidine and ketamine, were synthesized as intravenous anaesthetics with minimal impact on the cardiovascular or pulmonary system in 1956 and 1970, respectively. Initially, these agents were considered to be safe anaesthetics; however, those administrated with phencyclidine and ketamine exhibited psychotic side-effects, i.e., severe/prolonged delirium and schizophrenia-like psychosis [68,69]. Indeed, phencyclidine and ketamine generated schizophrenia-like psychosis in healthy individuals and aggravated symptoms of schizophrenics [15,16,17,24,68]. In contrast to NMDAR antagonists, two recent randomized, double-blind, placebo-controlled trials demonstrated that adjunctive therapy with benzoate, a D-amino acid oxidase inhibitor, improved cognitive function and the negative symptoms of patients with schizophrenia [70,71]. Inhibition of D-amino acid oxidase, which is a major degradation enzyme of D-serine in the central nervous system, increases D-serine levels [72,73]. Therefore, suppression of NMDAR function plays important roles in the pathophysiology of schizophrenia.

Contrary to these disadvantages of ketamine, the rapid acting antidepressant effects of ketamine in patients with major depression were demonstrated by placebo-controlled double-blind clinical trial in 2000 [8]. Other double-blind clinical studies also demonstrated that ketamine rapidly improved suicidal ideation compared with midazolam (active control) [74,75]. The responder ratio of ketamine was 25~85% within a day post-injection, and 14~70% at 3 days post-ketamine injection [21] (Table 1). In particular, 70.8% of antidepressant-resistant patients with depression improved by repetitive intravenous administration of ketamine (six times over 12 days) [76]. Therefore, compared to conventional monoaminergic antidepressants, in spite of severe psychotic side-effects, the surprisingly rapid action and efficacy of ketamine for the treatment of conventional antidepressant-resistant depression were demonstrated (Table 1).

Ketamine acutely produces various dose-dependent neuropsychiatric adverse effects [97]. Single intravenous administration commonly produces dissociation (distortions in visual, auditory, somatosensory stimuli and alterations in the perception of self or time), cognition (mental sharpness, concentration, recall, recognition, explicit and implicit), memory (vigilance, verbal fluency and delayed recalls), and positive (conceptual disorganization, hallucinations, suspiciousness, unusual thought content) and negative psychotomimetic effects (blunted affect, emotional withdrawal, motor retardation) [97]. Schizophrenia-like positive symptoms are considered to be due to the actions of esketamine rather than arketamine, since, using equimolar doses of esketamine and arketamine, esketamine was associated with acute psychotic reactions, but arketamine was not [98]. In contrast, arketamine contributes to relaxation and euphoric feelings [98]. Unfortunately, it has been speculated that repeated/sustained ketamine intake has been consistently associated with long-lasting memory deficits, and arketamine-induced euphoria is involved in the recreational feature of “kai-jai” [99].

It is well established that ketamine is a non-competitive NMDAR antagonist (Ki = 0.3~0.7 *μ*M), but it is also a high affinity dopamine D2 receptor partial agonist (Ki = 0.05~0.5 *μ*M) [100]. Although a recent meta-analysis reported that ketamine does not directly affect dopaminergic signalling [101], both clinical and preclinical findings show that the psychotomimetic effects of ketamine are mediated by its dopamine D2 receptor agonism [97,102,103,104]. Indeed, pre-administration of haloperidol prevented ketamine-induced agitation [104].

Contrary to the non-selective NMDAR antagonist ketamine, clinical trials reported the efficacy of the selective GluN2B antagonists CP-101,606 [95] and MK0657 [96] in the treatment of depressive states; however, the antidepressive effects of these selective GluN2B antagonists were comparatively modest and short-lived compared with that of ketamine (Table 1). In contrast, adverse psychiatric effects, including schizophrenia-like psychotic symptoms and dissociative responses, induced by CP-101,606 and MK0657 were lower than those induced by ketamine [95,96]. Until recently, in spite of the initial promising antidepressant potential of selective GluN2B antagonists, development of these compounds has been discontinued.

As shown Table 1, the clinical studies indicated consistent demonstrations that the noncompetitive NMDAR antagonists improved depression but aggravated schizophrenia or cognitive function; however, contrary to non-competitive NMDAR antagonists, several clinical studies reported that the enhancement of an endogenous NMDAR partial co-agonist, D-seine, improved both depression and schizophrenia (see details in review [105]). Traditionally, the efficacies of D-serine adjunctive therapy for schizophrenia had been studied by numerous clinical trials. Indeed, meta-analysis demonstrated that adjunctive D-serine modulation improved negative total symptoms of chronic schizophrenia [106]. Furthermore, inhibition of D-amino acid oxidase (major degradation enzyme of D-serine in the central nervous system [72,73]) improved cognitive function and the negative symptoms of patients with schizophrenia [70,71]. Contrary to schizophrenia, a randomized, double-blind, placebo-controlled trial reported that D-serine improved depressive mood in healthy volunteers [107]. This clinical evidence was supported by preclinical study using D-amino acid oxidase inhibitor [108]. These discrepancies between noncompetitive NMDAR antagonists and NMDAR co-agonists on schizophrenia and depressive disorders suggest that direct inhibition of cation channels in NMDAR and enhancement of GluN1 function are not clinically homologous. Elucidation of the underlying mechanism by which the GluN1 functional regulation affects mood and cognition possibly provides novel strategies for the development of novel therapeutic agents for treatment-resistant depression.

## 4. Preclinical Findings

### 4.1. Behavioural Study

Acute systemic administration of a non-competitive NMDAR antagonist increased locomotor activity and stereotypical behaviours in rodents [109,110,111,112]. NMDAR antagonism increases monoaminergic transmission, resulting in behavioural abnormalities [113,114], which are considered to be compatible with the positive symptoms of schizophrenia (Table 2) [115]. The stimulatory effect of non-competitive NMDAR antagonists on locomotor activity is enhanced by their long-term administration [110,111,112,116]. Non-competitive NMDAR antagonists generated severe disruptions in prepulse inhibition (PPI), and deficits in several domains of cognition, in rats (Table 2) [117,118]. Based on these functional changes following short- or long-term administration of NMDAR antagonists, it has been estimated that acute changes induced by NMDAR antagonists are comparable with those occurring in early stages of schizophrenia, but the duration of such changes induced by long-term administration appears to be more related to the persistence of clinical symptoms of schizophrenia [119,120,121,122,123].

Behavioural screening tests have provided important validation in the development of antidepressants [133]. Therefore, a novel screening framework is required for the development of novel effective antidepressants against conventional monoaminergic antidepressant-resistant depression. Paradoxically, utilizing animal models that do not respond to conventional monoaminergic antidepressants but are responsive to target agents that have shown efficacy in monoaminergic antidepressant-resistant patients with depression in the clinic can provide an improved framework to develop novel pharmacological screening for monoaminergic antidepressant-resistant depression (Table 2) [133]. Ideally, several animal models of monoaminergic antidepressant-resistant depression must be validated by demonstration that populations resistant to conventional monoaminergic antidepressants respond to medication that is effective in patients with depression [134]. Currently, some studies have focused on the understanding of which antidepressant responsiveness and resistance mechanisms are present in animal models [135]. According to these concepts, three basic approaches for the animal models of monoaminergic antidepressant-resistant depression have been proposed.

(1) Separation of rodents into bimodal subpopulations that respond or are resistant to traditional antidepressant treatments, which are often used following a behavioural stressor such as chronic mild stress [136] or chronic social defeat (Table 2) [137].
(2) Treatments that render rodents resistant to antidepressants (e.g., adrenocorticotropic hormone) [138] or inflammation [139] (Table 2).
(3) Genetic models that show resistance to conventional monoaminergic antidepressant treatments (e.g., use of genetically modified mice) (Table 2) [4,125].

Behavioural studies have demonstrated that non-competitive NMDAR antagonists exhibit antidepressant-like effects in forced swimming and tail suspension tests, in learned helplessness paradigms, and in animals exposed to chronic stressors [4,140,141,142,143]. Several studies reported that ketamine displayed rapid-acting antidepressant-like features in mice exposed to a learned helplessness paradigm and forced swimming test (Table 2) [4,124,125]. Several studies also demonstrated that ketamine produced antidepressant-like behaviour in animals exposed to various distinct stressors [127,144]. Furthermore, in the maternal deprivation protocol, ketamine could produce antidepressant-like effects in the forced swimming test (Table 2) [128,129,130].

The approval of esketamine has come with serious restrictions, since the doses of esketamine required for depression may cause dissociation and delirium, which probably presents shortly after onset of the drug but rapidly disappears just before the antidepressant response [8]. To overcome the adverse side effects, other NMDAR antagonism alternatives have been pursued. Selective antagonists to both GluN2A (NVP-AAM077) and GluN2B (Ro25–6981) have shown antidepressant-like effects without psychotomimetic-like activities preclinically [124,133,145]; however, combination administration of these two agents was sufficient to generate schizophrenia-like stereotypical behaviour [145].

Behavioural studies indicated that NMDAR inhibition probably contributed to the rapid-acting antidepressant effect but could not be involved in the long-lasting antidepressant effect. Indeed, the correlation between NMDAR binding affinity and antidepressant duration was not observed, since duration of the antidepressant effect of arketamine was longer than that of MK801 and esketamine, which show more potent affinity than arketamine (Table 2).

### 4.2. Signal Transduction Associated with NMDAR

Numerous investigations using depression rodent models have demonstrated that exposure to various stresses leads to enhancement of glutamatergic transmission and upregulation of NMDAR [53,146,147,148]. Chronic restraint stress increased the mRNA of GluN1, GluN2A and GluN2B in the hippocampus [149,150]. Maternal separation also increased the mRNA expression of GluN2A but not GluN2B in the hippocampus of adult rats [151]. Long-term administration of corticosterone, which mimics the endocrine response to stress, increased mRNA expression of GluN2A and GluN2B in the hippocampus [152]. Similar to the hippocampal response, a deficit of brain-derived neurotrophic factor (BDNF), which emulates the response to chronic stress and is considered to be a candidate mechanism of depression, also led to increased expression of GluN1, GluN2A and GluN2B mRNA in the frontal cortex during the early stage of development [153]. These findings suggest that upregulation of NMDAR induced by stress exposure and genetic abnormalities plays important roles in the pathomechanism of monoaminergic antidepressant-resistant depression, leading to the reasonable hypothesis that inhibition of NMDAR signalling contributes to robust antidepressant-like action.

Neither acute nor chronic administration of ketamine affected serum levels of corticosterone and adrenocorticotropic hormone (ACTH), whereas administration of both prevented elevation of corticosterone and ACTH levels induced by chronic mild stress [127]. In contrast, BDNF expression in the hippocampus was not affected by mild chronic stress or ketamine [127]. Both ketamine and MK801 increased BDNF expression in the frontal cortex but not the nucleus accumbens [4].

Acute MK801 administration downregulated hippocampal GluN1 and GluN2B [154], whereas contrary to NMDAR, the GluA1 subunit of the α-amino-3-hydroxy-5-methyl-4-isoxazolepropionic acid (AMPA)/glutamate receptor (AMPAR) was increased by ketamine [133,155,156,157]. The mechanisms of ketamine-induced upregulation of GluA1 are speculated to activate mammalian target of rapamycin (mTOR) [133,158] and/or extracellular signal-regulated kinase (Erk) signalling [159], which regulates the initiation of protein translation, and protein synthesis, for synaptogenesis [160,161]. mTOR signalling plays a responsible role in the antidepressant effects of ketamine, since local administration of rapamycin (mTOR inhibitor) into the medial prefrontal cortex prevented the antidepressant effects of ketamine [133]. In spite of these efforts, enhanced mTOR and Erk signalling can explain the increased AMPAR expression, but contradicts the decreased NMDAR expression. 

### 4.3. Neurotransmitter Release Associated with NMDAR

**Figure 1:**
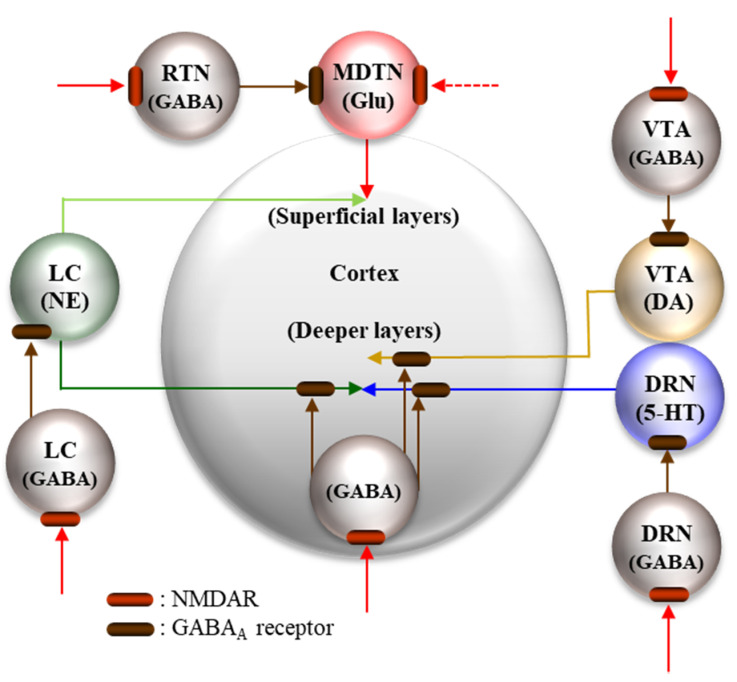
Schematic regulation mechanisms associated with NMDAR in neural circuits of dopaminergic (DA), serotonergic (5-HT), noradrenergic (NE) and glutamatergic (Glu) pathways to the frontal cortex (medial prefrontal cortex, insular cortex and orbitofrontal cortex). Dopaminergic (from ventral tegmental area: VTA), serotonergic (from dorsal raphe nucleus: DRN) and noradrenergic (from locus coeruleus: LC) neurones project their terminals to deeper layers of the frontal cortex, and receive regional GABAergic inhibition, which is regulated by stimulatory NMDAR. Contrary to the monoaminergic mesocortical pathway, glutamatergic neurones project terminal from the mediodorsal thalamic nucleus (MDTN) to superficial layers of the frontal cortex. Glutamatergic neurones in the MDTN receive intrathalamic GABAergic inhibition mainly from the reticular thalamic nucleus (RTN) and the MDTN, which are also regulated by NMDAR. Yellow, blue, deep green, red and brown arrows indicate the projection terminals of DA, 5-HT, LC, GABA and Glu, respectively. Light green arrow indicates the catecholaminergic co-releasing projection (NE plus DA).

Systemic administration of NMDAR antagonists acutely increased the release of glutamate [34,37,162], dopamine [34], norepinephrine [34,163] and serotonin (5-HT) [164] in the frontal cortex. Inhibition of NMDAR in the frontal cortex increased regional monoamine levels [39,40,41,42,165] (Figure 1). Inhibition of NMDAR in the ventral tegmental area (VTA), LC and dorsal raphe nucleus (DRN) also increased respective dopamine, norepinephrine and 5-HT releases in the frontal cortex [32,33,34,35,36,39,40,166,167] (Figure 1). This release of monoamines in the frontal cortex induced by systemic NMDAR antagonists is generated by cortical and sub-cortical GABAergic disinhibition, since GABA_A_ receptor agonist prevented this monoamine release [32,33,34,35,36,39,40,166,167] (Figure 1). Contrary to monoamines, inhibition of NMDAR in the frontal cortex did not affect regional L-glutamate release, whereas inhibition of NMDAR in the mediodorsal (MDTN) and reticular thalamic nuclei (RTN) drastically increased L-glutamate release in the frontal cortex [29,32,34,36,37,38,166]. Activation of the GABA_A_ receptor in MDTN or RTN suppressed L-glutamate release in the frontal cortex induced by NMDAR inhibition in the thalamus [32,34,36,37,162,166]. The L-glutamate release in the frontal cortex induced by systemic NMDAR antagonist administration is generated by thalamic GABAergic disinhibition, but not by frontal GABAergic disinhibition (Figure 1). Previous microdialysis studies suggest that a deficit of NMDAR in sub-cortical neural circuits increases neurotransmitter release in the frontal cortex via GABAergic disinhibition.

Taken together with the recent findings that enhanced GABAergic transmission is associated with parvalbumin-expressing interneurones [168,169,170] and upregulation of NMDAR [53,146,147,148], the compounds that can establish GABAergic disinhibition of parvalbumin-expressing GABAergic interneurones by targeting the microcircuit between glutamatergic and GABAergic transmission systems hold promise as rapid-acting antidepressants and represent a breakthrough strategy for the treatment of depression.

## 5. Candidate Pathophysiology of Depression and Schizophrenia Associated with NMDAR

### 5.1. Molecular Mechanism

The upregulation and downregulation of NMDAR expression in depression and schizophrenia, respectively, were observed in post-mortem studies [54,55,60,61,62,63,64,65]. Taken together with the clinical evidence that a non-competitive NMDAR antagonist, ketamine, generates schizophrenia-like psychotic symptoms and improves depression, the functional abnormalities of glutamatergic transmission associated with NMDAR probably contribute to the pathomechanisms of schizophrenia and depression. Other studies have reported that chronic exposure to stress enhanced parvalbumin-expressing GABAergic interneurones underlies depression-like behaviour [168,169,170]. Numerous microdialysis studies have also demonstrated that NMDAR antagonists exert a preferential suppression of NMDAR on GABAergic interneurones, resulting in enhanced neurotransmitter release in the frontal cortex via GABAergic disinhibition [29,34,35,36,37,39,41,42,162,165,166,171,172]. A recent study using the conditional knockdown technique clearly demonstrated that selective knockdown of GluN2B on somatostatin or parvalbumin expressing GABAergic interneurones blocked the antidepressant-like action of ketamine, whereas that on glutamatergic pyramidal neurones was not affected [173]. Therefore, the suppression of NMDAR function on the enhanced GABAergic interneurones plays fundamental roles in the improvement of several disturbances in the pathomechanisms associated with depression. Considered with the demonstrations of two clinical trials that GluN2B selective antagonists (CP-101,606 and MK0657) did not have rapid-acting antidepressant effects and exhibited fewer neuropsychiatric adverse effects [95,96], rapid-acting antidepressant effects probably require broad inhibition of NMDAR containing both GluN2A and GluN2B on GABAergic interneurones.

### 5.2. Pathophysiological Neural Circuits

Psychiatric disorders such as schizophrenia and depression are possible human-specific diseases in that they are diagnosed using various interview techniques with communication tools. In other words, it is not possible to verify the validity of animal models that reproduce the psychiatric dysfunction of schizophrenia or depression. Acute systemic administration of non-competitive NMDAR antagonists leads to hyperlocomotion and stereotypical behaviours in rodents that are potentially compatible with the positive symptoms of schizophrenia [109,115]. NMDAR antagonists also produce disruptions in PPI, and several deficits in several distinct domains of cognition in rodents [117,118]. The pathomechanisms of PPI deficit has yet to be clarified, whereas several studies using functional magnetic resonance imaging (fMRI) suggested that disturbance of the MDTN, including the cortico-pallido-thalamic pathway, plays a key role in the PPI disruption [174,175]. Another fMRI study reported that ketamine supressed the functional connectivity between the LC and MDTN [176]. Therefore, NMDAR may contribute to the efficient switching between hippocampus-dependent and hippocampus-independent learning processes [177], leading to the regulation of attention and memory consolidation/reconsolidation processes [32,34,35,36,37,38,162,166,178,179].

Traditionally, the function of the MDTN on cognition has been considered to be mapped in specific memory recognition and exclusive cognitive domains [180]; however, abundant recent evidence indicates that several neural circuits including the MDTN are involved in several neuropsychiatric abnormalities in which the cognitive impairments are not restricted to memory functions [181]. In particular, it has been widely shown that the MDTN receives various inputs from the amygdala, and the cortical and subcortical regions associated with learning, memory, emotion, and perceptual integration [182,183,184], but the MDTN is mainly regulated by GABAergic inhibition from the RTN, which is activated by noradrenergic input from the LC, and directly receives serotonergic input from the DRN [32,34,35,36,38,166,178,185]. The MDTN projects glutamatergic terminals to various cortical regions such as mPFC, insula, orbitofrontal cortex (OFC) and basal ganglia [32,33,34,35,36,37,41,42,162,166,171,178,179,186,187,188]. These thalamocortical pathways have mainly been explored through experiments in rodents, primates and humans, and these functional interpretations of the cognitive mechanisms have been shown to translate from rodents to humans [180] (but this pathway is speculated to be weak in rodents [189]). This section tries to discuss how such neuroscientific understanding will propel future efforts into development of a rapid-acting antidepressant class that does not result in cognitive dysfunction.

#### 5.2.1. Supressive Regulation of Enhanced Thalamocortical Glutamatergic Transmission

Inhibition of NMDAR in RTN and MDTN enhanced thalamocortical glutamatergic transmission from the MDTN to mPFC, insula and OFC [32,33,34,35,37,162,166]. Glutamatergic neurones in the MDTN receive postsynaptic excitatory 5-HT receptor type 7 (5-HT7R) [34,35] and extrasynaptic group II metabotropic glutamate receptors (II-mGluR) [37,162]. Glutamatergic terminals in the frontal cortex receive inhibitory II-mGluR and group III metabotropic glutamate receptors (III-mGluR) presynaptically [36,37,162,166].

Hyperactivation of thalamocortical glutamatergic transmission due to intrathalamic GABAergic disinhibition was supressed by several atypical antipsychotics: aripiprazole through enhancement of II-mGluR [162], clozapine through enhancement of III-mGluR [166,190], and lurasidone through inhibition of postsynaptic 5-HT7R [34,35]. Interestingly, the suppression of thalamocortical glutamatergic transmission associated with II-mGluR and III-mGluR were possibly mediated by astroglial transmission but not by neurotransmission, since astroglial release of L-glutamate from system Xc^-^ and hemichannel stimulated II-mGluR and III-mGluR [36,37,166,191]. Both memantine and N-acetyl-L-cysteine inhibited MK801-induced L-glutamate release in the frontal cortex (thalamocortical glutamatergic transmission) via activation of astroglial L-glutamate release through system Xc^-^ [37,162,166]. Indeed, behavioural deficits in rat phencyclidine models are addressed by N-acetyl-L-cysteine administration [192].

Both systemic administration and local administration into the MDTN of a 5-HT transporter inhibitor increased extracellular 5-HT level in the MDTN, resulting in partially enhanced thalamocortical glutamatergic transmission [33,34,35]. The 5-HT7R antagonists, SB266970 and lurasidone, compensate for hyperactivation of glutamatergic transmission induced by enhanced serotonergic transmission [33,34,35]. Pharmacological behavioural preclinical studies demonstrated that SB266970 has an antipsychotic action with rapid-acting antidepressant effects, as opposed to conventional monoaminergic antidepressants, and it augments the actions of conventional monoaminergic antidepressants [193]. In addition to the acute effects of 5-HT7R antagonists, 5-HT7R signalling contributes to synapse remodelling and neural network formation, which is the target of event-related structural and functional plasticity. However, after maturation of neural circuits during adolescence and adulthood, 5-HT7R inhibition provides the protection of generation of abnormal neural circuits induced by chronic exposure to severe stress via preventions of disruption and/or regeneration [34,35]. The effects of serotonin receptor type 1A (5-HT1AR) and 5-HT7R are opposite in regard to neurotransmission and cognition [34,35,194], but the effect of 5-HT1AR is predominant rather than that of 5-HT7R, resulting in insufficient understanding of the function of 5-HT7R in psychopharmacology. Although combination therapy between SSRI/SNRI and ketamine/esketamine [81,90] has been studied, the interaction between 5-HT7R antagonistic agents and NMDAR antagonists in treatment-resistant depression represents a novel aspect of the development of rapid-acting antidepressants.

#### 5.2.2. Stimulatory Regulation of Enhanced Thalamocortical Glutamatergic Transmission

Contrary to the NMDAR antagonist schizophrenia model, it is well known that dysfunction of MDTN plays a role in the cognitive dysfunction of ADHD, autism and intellectual disability [195,196,197]. Thalamocortical glutamatergic transmission is impaired in experimental animal models of ADHD and autism [178,179,187,188]. Physiological activation of the thalamic activity reduced distraction in attention tests [198], whereas the pathological enhancement of the thalamocortical glutamatergic transmission induced by phencyclidine disturbs working memory, which is compensated by a therapeutic-relevant dose of guanfacine [199].

**Figure 2:**
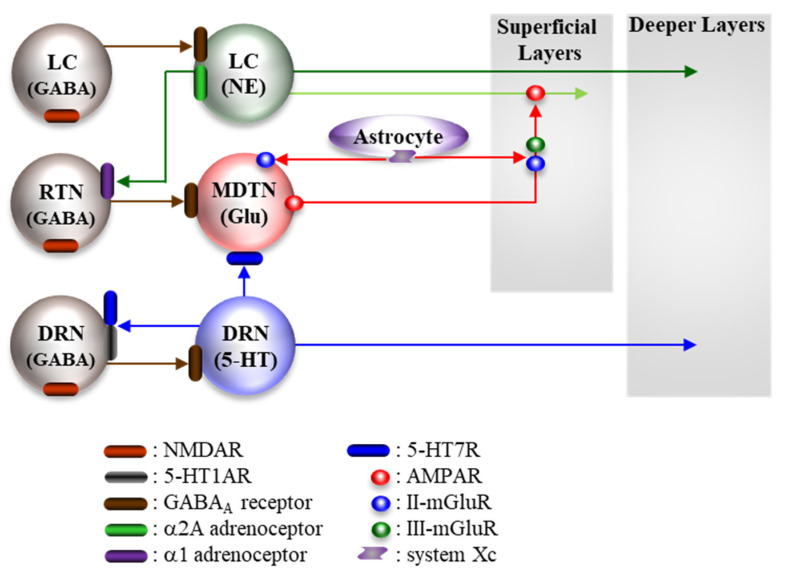
Proposed hypothesis for the extended complicated neural circuit connectivities involved in the thalamocortical cognitive glutamatergic pathway, from the MDTN to the frontal cortex; the mesothalamic serotonergic pathway, from the DRN to the MDTN; the mesothalamic noradrenergic pathway, from LC to the RTN; the mesocortical catecholaminergic pathway, from the LC to the frontal cortex; the mesocortical serotonergic pathway from the DRN to the frontal cortex. Generally, both noradrenergic and serotonergic neurones project selective terminals to deeper layers of the frontal cortex; however, some neurones in the LC project catecholaminergic co-releasing terminal (co-releasing norepinephrine with dopamine) to superficial layers of the frontal cortex. Glutamatergic projection from the MDTN presynaptically activates catecholaminergic co-releasing terminals via AMPAR in the superficial layers of the frontal cortex. Glutamatergic neurones in the MDTN receive excitatory serotonergic input from DRN via 5-HR7R and inhibitory GABAergic inhibition from RTN. GABAergic neurones are regulated by excitatory NMDAR and receive excitatory noradrenergic input from LC via α1 adrenoceptors.

Catecholaminergic neurones in the LC project at least three terminals; selective noradrenergic terminals to deeper layers of the frontal cortex [41,42,200,201] and RTN [32,37], and catecholaminergic co-releasing terminal (co-releasing norepinephrine and dopamine) to the superficial layers of the frontal cortex [41,42,200,201]. GABAergic neurones in the RTN receive excitatory noradrenergic input from the LC via the α1 adrenoceptor [32,178]. The intrathalamic GABAergic pathway is regulated by the inhibitory presynaptic α2A adrenoceptor in the MDTN [32,178]. Therefore, systemic administration of guanfacine supresses GABAergic inhibition of glutamatergic neurones in the MDTN via activation of the α2A adrenoceptor in the LC and MDTN, resulting in enhanced thalamocortical glutamatergic transmission [178]. If the hyperfunction of thalamocortical glutamatergic transmission contributes to the fundamental mechanisms of NMDAR antagonist-induced cognitive deficit, the suppressive effects of guanfacine on GABAergic inhibition in the MDTN apparently aggravate NMDAR antagonist-induced cognitive deficits. The discrepancy between NMDAR antagonists and guanfacine on cognition leads us to hypothesise that the regulation mechanisms in GABAergic inhibition of cognition are important. GABAergic disinhibition induced by NMDAR inhibition and α2A adrenoceptor activation generate tonic/persistent and phasic/transient GABAergic disinhibition, respectively [32,34,178] (Figure 2). Therefore, tonic GABAergic disinhibition by NMDAR inhibition abolishes input signalling from other regions via continuous hyperactivation, whereas, conversely, phasic GABAergic disinhibition by α2A adrenoceptor activation possibly leads to input optimization [32,36,37].

## 6. Conclusions and Remaining Challenges

Numerous clinical and preclinical investigations have already provided us with a glimpse into the several candidate pathomechanisms of the various conditions behind conventional monoaminergic antidepressant-resistant depression. There is no doubt that inhibition of upregulated NMDAR on GABAergic interneurones induced by chronic exposure to stress is one of the pragmatic mechanisms of the improvement of conventional monoaminergic antidepressant-resistant depression. Current findings show the possible potential of NMDAR inhibition, where it, at least partially, improves dysfunction of emotional perception/cognition (anhedonia and suicidal ideation) and displays rapid-acting antidepressant effects in conventional monoaminergic antidepressant-resistant depression.

The majority of individuals that take ketamine/esketamine have to overcome various dose-dependent dissociation cognitive deficits and psychotomimetic responses before getting the excellent beneficial clinical effects of ketamine. Furthermore, the antidepressant effect of ketamine/esketamine is comparatively short-lived (can last 1~2 weeks), whereas individuals who positively respond to ketamine usually relapse [80,88]. Therefore, NMDAR-inhibiting antidepressive medication is a double-edged sword therapy that carries the risk of severe persistent schizophrenia-like psychosis and long-lasting memory/cognitive deficits induced by chronic/repeated intake of an NMDAR antagonist. Unfortunately, the threshold dosage for antidepressive and psychotomimetic actions of ketamine is almost equal (cannot be discriminated).

The interpretation of the various pharmacological mechanisms of rapid-acting antidepressant effects and the neuropsychiatric adverse response of ketamine, obtained from both clinical and preclinical investigations, is not of any pharmacological value unless a critical separation between their neuropharmacological processes can be understood. In spite of the potential rapid-acting antidepressive, anti-anhedonia and anti-suicidal ideational actions of NMDAR antagonists, several challenges remain to be resolved to produce effective clinical applications of NMDAR antagonists for treatment in conventional monoaminergic antidepressant-resistant depression. Finally, we summarize the remaining challenging targets to progress rapid-acting antidepressive therapy.

1. Ideally, when a clear pharmacodynamic/pharmacokinetic distinction between the antidepressive and neuropsychotomimetic effects of NMDAR antagonists is achieved, then, according to the novel strategy, we can develop a new class of rapid-acting antidepressant for treatment of conventional monoaminergic antidepressant-resistant depression.
2. Unfortunately, current findings suggest the induction mechanisms of NMDAR antagonists associated with antidepressive and neuropsychotomimetic effects are possibly identical. Therefore, it is important to identify the strategy of adjunctive therapies that gives antipsychotic effects without affecting the antidepressant effects of NMDAR antagonists.
3. The high affinity dopamine D2 receptor partial agonistic action of ketamine possibly contributes to either its antidepressive or neuropsychotomimetic actions. Therefore, determination of the effects of adjuvant medication (typical and atypical antipsychotics) on the antidepressive and neuropsychotomimetic effects of ketamine is a rational strategy for the rapid-acting monoaminergic antidepressant-resistant depression therapy.
4. If these above trials do not show beneficial outcomes, we should explore other neuromodulation therapies for prevention of the acute and chronic adverse effects of ketamine without affecting its antidepressive action.
5. Preclinical findings suggest that distinct hippocampal and thalamic non-dopaminergic mechanisms play important roles in the ketamine-induced cognitive/memorial deficits. Thalamic nuclei that receive various inputs from cortical and subcortical regions integrate to give precise output to the frontal cortex. Therefore, conversion from tonic activation of thalamic activity induced by NMDAR inhibition to phasic activation/inhibition can lead to the development of cognitive promoting medication.

## Figures and Tables

**Table 1:** Completed double-blind, placebo-controlled trials assessing ketamine and other putative N-methyl-D-aspartate/glutamate receptor (NMDAR) antagonists. The present study searched MEDLINE using the keywords “ketamine”, “depression” and “randomized controlled trial” until January 1st, 2020. Relevant articles were obtained in full and assessed for inclusion independently by reviewers. The disagreement among reviewers was resolved via discussion to reach consensus. The reports that indicated the responder ratios are shown in Table 1.

Drug	Regimen	Diagnosis	Placebo (N)	Outcome Responder Ratio [Drug vs. Placebo]	Reference
**Ketamine**					
Double-blind	0.5 mg/kg (40 min) single iv	Major and bipolar depression	Saline (9)	Reduced HDRS 240 min (initial) 72 h (sustain)	[8]
Double-blind	0.5 mg/kg (40 min) single iv	Major depression	Propofol/fentanyl (70)	Reduced HDRS 24 h [71% vs. 0%]	[7]
Double-blind	0.5 mg/kg (40 min) single iv	Treatment-resistant depression	Saline (18)	Reduced HDRS 110 min (initial) 7 days (sustain) [71% vs. 0%]	[7]
Double-blind (added on mood stabilizer)	0.5 mg/kg (40 min) single iv (maintained Li or VPA)	Treatment-resistant bipolar depression	Saline (18)	Reduced MADRS 40 min (initial) 3 days (sustained) [71% vs. 6%]	[77]
Double-blind (added on mood stabilizer)	0.5 mg/kg (40 min) single iv (maintained Li or VPA)	Treatment-resistant bipolar depression	Saline (15)	Reduced MADRS 40 min (initial) 3 days (sustained) [71% vs. 0%]	[78]
Double-blind	0.5 mg/kg (40 min) single iv	Treatment-resistant depression	Midazolam (73)	Reduced MADRS 24 h (initial) 7 days (sustained) [64% vs. 28%]	[74,79]
Double-blind	50 mg intranasal administration	Major depression	Saline (20)	Reduced MADRS 24 h (initial) 7 days (sustain)	[80]
Double-blind (added on SSRI)	0.5 mg/kg (40 min) single iv	Major depression	Saline (30)	Reduced MADRS 2 h min (initial) [92% vs. 57%]	[81]
Double-blind	0.5 mg/kg (40 min) 2~3 times iv over 15 days	Treatment-resistant depression	Saline (67)	Reduced MADRS 7 days (initial) 15 days (sustain) [69% vs. 9%]	[5]
Double-blind	0.2, 0.5 mg/kg (40 min) single iv	Treatment-resistant depression	Saline (64)	Reduced HDRS 40 min (initial) [25% vs. 0%]	[82]
Double-blind	0.2, 0.5 mg/kg (40 min) single iv	Treatment-resistant depression	Saline (95)	Reduced HDRS 40 min (initial) 28 days (sustain) [46% vs. 13%]	[83]
Double-blind	0.5 mg/kg (40 min) single iv	Treatment-resistant bipolar depression	Midazolam (16)	Reduced HDRS 24 h (initial) [89% vs. 0%]	[84]
Double-blind	0.5 mg/kg (40 min) single iv	Treatment-resistant depression	Midazolam (80)	Reduced HDRS 24 h (initial) [30% vs. 15%]	[75]
Double-blind	0.1, 0.2, 0.5, 1.0 mg/kg (40 min) single iv	Treatment-resistant depression	Midazolam (99)	Reduced HDRS 24 h (initial) 21 days (sustain) [57% vs. 33%]	[85]
Double-blind	0.5 mg/kg (40 min) 6 times iv over 14 days	Treatment-resistant depression	Midazolam (41)	Reduced MADRS 24 h (initial) 7 days (sustained) [59%]	[86]
Double-blind	0.5 mg/kg (45 min) 6 times iv over 21 days	Treatment-resistant depression	Saline (26)	Reduced HDRS 21 days (sustain) [25% vs. 33%]	[87]
**Esketamine**					
Double-blind	0.2 or 0.4 mg/kg single iv	Treatment-resistant depression	Saline (29)	Reduced MADRS 2 h (initial) 35 days (sustain) [64% vs. 0%]	[88]
Double-blind	28, 56, 84 mg intranasal administration	Treatment-resistant depression	Simulated placebo of esketamine taste (denatonium benzoate) (126)	Reduced MADRS 2 h (initial) 74 days (sustain) [50% vs. 10%]	[89]
Double-blind	84 mg intranasal administration	Treatment-resistant depression	Simulated placebo of esketamine taste (66)	Reduced MADRS 4 h (initial) 25 days (sustain) [50% vs. 10%]	[90]
Double-blind (added on SSRI or SNRI)	56, 84 mg intranasal administration	Treatment-resistant depression	Simulated placebo of esketamine taste (197)	Reduced MADRS 24 h (initial) 74 days (sustain) [69.3% vs. 52%]	[91]
Double-blind (added on SSRI or SNRI)	56, 84 mg intranasal administration (twice a week for 4 weeks)	Treatment-resistant depression	Simulated placebo of esketamine taste (346)	Reduced MADRS 24 h (initial) 28 days (sustain) [53.1% vs. 38.9%]	[92]
Double-blind	Esketamine (0.25 mg/kg, 40 min, single iv)	Treatment-resistant depression	Ketamine (0.5 mg/kg, 40 min, single iv) (63)	Reduced MADRS 24 h (initial)7 days [43.7% vs. 62.1%]	[93]
Double-blind (added on SSRI or SNRI)	28, 56, 84 mg intranasal administration (twice a week for 4 weeks)	Treatment-resistant depression (>65 years old)	Simulated placebo of esketamine taste (denatonium benzoate) (137)	Reduced MADRS 28 days (sustain) [27.0% vs. 13.3%]	[94]
**CP-101,606**					
Double-blind (added on paroxetine)	0.75 mg/kg CP-101,606 (90 min) 2 times iv for 6.5 h	Paroxetine-resistant major depression	Saline (30)	Reduced HDRS 2 days (initial) 8 days (sustain) [60% vs. 20%]	[95]
**MK-0657**					
Double-blind	4 mg/day (po) increased 4, 8, 12 mg/day until 12 days	Treatment-resistant depression	Saline (5)	Reduced HDRS 5 days (initial) 12 days (sustain)	[96]

**Table 2:** Behavioural study assessing ketamine and other agents.

Model	Agent	Effect	Reference
**Schizophrenia**			
locomotor activity stereotypical behaviour	MK801 Phencyclidine	hyperlocomotion	[109] [110,111,112]
prepulse inhibition (PPI)	MK801 Phencyclidine	disruptions	[117,118]
**Depression**			
learned helplessness	Ketamine	rapid acting antidepressant effect	[4,124]
forced swimming	Ketamine MK801 Ro25-6981 CPP Imipramine fluoxetine NBQX	rapid acting antidepressant effect rapid acting antidepressant effect rapid acting antidepressant effect rapid acting antidepressant effect no antidepressant effects no antidepressant effects no antidepressant effects (supress antidepressant effects of ketamine, MK801 and Ro25-6981)	[4,124,125] [4,124,126] [124] [4,125] [4] [4] [124]
sucrose consumption (anhedonia test) (after chronic mild stress)	Ketamine	no antidepressant effects antidepressant/antianhedonic effect antidepressant effect	[4] [127] [4,127]
novelty-suppressed feeding (after chronic mild stress)	Ketamine	no antidepressant effects antidepressant effect	[4] [4]
fear conditioning	ketamine	No effect	[4]
passive avoidance tests	ketamine	not impair fear memory retention.	[124]
maternal deprivation	ketamine	antidepressant effect	[128,129,130]
TrkB knockout forced swimming novelty-suppressed feeding	Ketamine, MK801 ketamine	no antidepressant effects no antidepressant effects	[4] [4]
BDNF knockout Forced swimming	Ketamine MK801	no antidepressant effects no antidepressant effects	[125] [4]
**Arketamine/Esketamine**	**Arketamine**	**Esketamine**	
learned helplessness	rapid acting antidepressant effect	no antidepressant effect	[131]
forced swimming	rapid acting antidepressant effect longer-lasting antidepressant effect than esketamine	rapid acting antidepressant effect	[132]
tail suspension	rapid acting antidepressant effect longer-lasting antidepressant effect than esketamine	rapid acting antidepressant effect	[132]
social defeat stress	rapid acting antidepressant effect longer-lasting antidepressant effect than esketamine	rapid acting antidepressant effect	[131]
repeated corticosterone	rapid acting antidepressant effect longer-lasting antidepressant effect than esketamine	rapid acting antidepressant effect	[132]

## Abbreviations

II-mGluR	group II metabotropic glutamate receptors
III-mGluR	group III metabotropic glutamate receptors
5-HT	serotonin
5-HT2AR	serotonin receptor type 2A
5-HT7R	serotonin receptor type 7
ACTH	adrenocorticotropic hormone
AMPA	α-amino-3-hydroxy-5-methyl-4-isoxazolepropionic acid
AMPAR	α-amino-3-hydroxy-5-methyl-4-isoxazolepropionic acid/glutamate receptor
BDNF	brain-derived neurotrophic factor
DRN	dorsal raphe nucleus
Erk	extracellular Signal-regulated Kinase
EMA	the European Medicines Agency
FDA	the Food and Drug Administration
fMRI	functional magnetic resonance imaging
GABA	γ-aminobutyrate
LC	locus coeruleus
MDTN	mediodorsal thalamic nucleus
MK801	dizocilpine
mTOR	mammalian target of rapamycin
NMDAR	N-methyl-D-aspartate/glutamate receptor
OFC	orbitofrontal cortex
PPI	prepulse inhibition
RTN	reticular thalamic nucleus
SNRI	serotonin norepinephrine reuptake inhibitor
SSRI	selective serotonin reuptake inhibitor
VTA	ventral tegmental area
